# Immune response of horses to inactivated African horse sickness vaccines

**DOI:** 10.1186/s12917-020-02540-y

**Published:** 2020-09-01

**Authors:** Marina Rodríguez, Sunitha Joseph, Martin Pfeffer, Rekha Raghavan, Ulrich Wernery

**Affiliations:** 1 Central Veterinary Research Laboratory, P.O. Box 597, Dubai, UAE; 2 Veterinary Faculty, University of Leipzig, Leipzig, Germany

**Keywords:** African horse sickness, Immune response, Inactivated vaccine

## Abstract

**Background:**

African horse sickness (AHS) is a serious viral disease of equids resulting in the deaths of many equids in sub-Saharan Africa that has been recognized for centuries. This has significant economic impact on the horse industry, despite the good husbandry practices. Currently, prevention and control of the disease is based on administration of live attenuated vaccines and control of the arthropod vectors.

**Results:**

A total of 29 horses in 2 groups, were vaccinated. Eighteen horses in Group 1 were further divided into 9 subgroups of 2 horses each, were individually immunised with one of 1 to 9 AHS serotypes, respectively. The eleven horses of Group 2 were immunised with all 9 serotypes simultaneously with 2 different vaccinations containing 5 serotypes (1, 4, 7–9) and 4 serotypes (2, 3, 5, 6) respectively. The duration of this study was 12 months. Blood samples were periodically withdrawn for serum antibody tests using ELISA and VNT and for 2 weeks after each vaccination for PCR and virus isolation. After the booster vaccination, these 27 horses seroconverted, however 2 horses responded poorly as measured by ELISA. In Group 1 ELISA and VN antibodies declined between 5 to 7 months post vaccination (pv). Twelve months later, the antibody levels in most of the horses decreased to the seronegative range until the annual booster where all horses again seroconverted strongly. In Group 2, ELISA antibodies were positive after the first booster and VN antibodies started to appear for some serotypes after primary vaccination. After booster vaccination, VN antibodies increased in a different pattern for each serotype. Antibodies remained high for 12 months and increased strongly after the annual booster in 78% of the horses. PCR and virus isolation results remained negative.

**Conclusions:**

Horses vaccinated with single serotypes need a booster after 6 months and simultaneously immunised horses after 12 months. Due to the non-availability of a facility in the UAE, no challenge infection could be carried out.

## Background

African horse sickness (AHS) is an insect-borne viral disease of equids that is endemic to sub-Saharan African countries [1, 2]. The disease can be acute, subacute or subclinical but is usually characterised by clinical signs and lesions associated with respiratory and circulatory impairment [2]. The disease appears in 4 classical forms: pulmonary, cardiac, and mixed pulmonary and cardiac forms and horse sickness fever [3]. The mixed, often acute form is most commonly observed. The fourth form, horse sickness fever, is often overlooked because it is a mild form and seen in least susceptible equids such as donkeys and zebras [1] and sometimes in horses immunised with inactivated vaccines (Wernery, 2019, pers. communication). AHS is caused by African horse sickness virus (AHSV) of the genus *Orbivirus* in the family *Reoviridae*. Biting midges (*Culicoides* spp.) are the principal vectors, and *C. imicola* is the most important midge for AHSV transmission [4], but *C. bolitinos* also plays an important role. The virus has been isolated from the dog tick *Rhipicephalus sanguineus* [5] and the camel tick *Hyalomma dromedarii* [6]. However, ticks and mosquitoes do not play an important role in the epidemiology of AHS. Wet climatic conditions favour *Culicoides* biting midges for the transmission of the virus and their expansion northwards into the Mediterranean Basin of Europe. This is of great concern for AHS outbreaks in Europe similar to the recently experienced outbreaks with bluetongue virus (BTV) [7]. To date, 9 immunologically distinct serotypes (1 to 9) have been identified, and all 9 serotypes exist in sub-Saharan Africa and East Africa. AHS serotypes 2, 4 and 9 have been confirmed to circulate in North and West Africa, where they are occasionally experienced in Mediterranean countries. Outside Africa, AHS outbreaks have been documented in the Middle East (1959–1963), Spain (serotype 9 in 1966; serotype 4 in 1987–1990) and Portugal (serotype 4 in 1989) [8]. During the period of 1959–1961, the disease even spread as far as Pakistan and India, causing fatalities of approximately 300,000 equids [2, 9]. In 2007, an AHS serotype 2 epidemic occurred in Senegal with 232 outbreaks and 1137 horse fatalities [7]. In April 2019, another AHS outbreak occurred in Chad, causing a fatality rate of 85.11% (https://www.oie.int/wahis_2/public/wahid.php/Reviewreport/Review?page_refer=MapFullEventReport&reportid=30236) and February 2020 in Thailand (https://www.oie.int/wahis_2/public/wahid.php/Reviewreport/Review?page_refer=MapFullEventReport&reportid=33912).

Host species for the AHSV are equids, dogs, elephants, camels, cattle, sheep, goats, and predatory carnivores (by eating infected meat) [10]. The disease affects mainly equids, with horses being most susceptible to AHS with a mortality rate of 50–95%, followed by mules with mortality of approximately 50%. Donkeys are least susceptible to AHS and experience only subclinical infections [8]. The infection in zebras is mostly asymptomatic [11]; however, they may develop fever and viremia for up to 40 days. Zebras are frequently implicated as the cause of AHS outbreaks, but this is most likely a misconception. Zebras have no significant role in the epidemiology of AHSV, as AHS outbreaks are also reported in areas where zebras do not exist. Moreover, AHS outbreaks start in areas of high horse density where zebras are not necessarily present [9]. Canines are known to contract the severe form of AHS by eating contaminated horse meat but were thought to be ‘dead-end’ hosts of the virus. New research, however, indicates that domestic dogs could play a role in the transmission of AHSV, as it was shown that dogs become infected not only by consuming contaminated meat but also by transmission through the vector. Nevertheless, there is no definitive proof that dogs can transmit the virus to midges [12, 13].

The first attempts to control AHS by vaccination date back to the middle of the last century by using an available live-attenuated vaccine, which even today provides strong humoral and cellular immunity. However, studies revealed a possible inherent risk associated with this vaccine by reverting to virulence and subsequent disease spread.

Gene segment reassortment between vaccine and field serotypes and reversion to virulence of live attenuated vaccine viruses account for such shortcomings of live attenuated vaccines [14]. Among the alternative vaccine candidates which are sub unit vaccines, DNA vaccines, reverse genetic vaccines, inactivated vaccines are considered safe but are uneconomical and can only induce protective immunity upon multiple administrations [11, 15]. Therefore, we developed inactivated vaccines from serotypes isolated from horse fatalities in Kenya, where all 9 serotypes circulate [16]. Recently, a public announcement to horse owners in South Africa was made regarding a new vaccine referred to as “DCA Vac”. This vaccine is an inactivated vaccine containing 8 serotypes, with serotype 5 not being included.

The aim of this vaccination experiment was to evaluate the serological response in AHS-naïve horses after they were vaccinated with inactivated AHS vaccines containing all 9 serotypes. The results may lead to the production of safe and effective inactivated AHS vaccines that protect equids against the disease before modern recombinant subunit AHS vaccines become a reality.

## Results

### Group 1: antibody results in 18 horses vaccinated with a single serotype 1 to 9

Before vaccination, sixteen horses were negative by both tests, whereas 2 horses, 5 and 11, showed positive competitive enzyme-linked immunosorbent assay (cELISA) results with a percentage of inhibition (PI) of 68.5 and 57.0%, respectively, and virus neutralisation (VN) titres for both were between 2 and 3.75 against 7 serotypes with no antibodies detected against 2 serotypes (6 and 9). Both animals had been vaccinated 10 years ago with the live attenuated Onderstepoort vaccine (OBP LAV). PCR and virus isolation were performed regularly for 2 weeks after each vaccination using EDTA blood and tested negative.

**Fig. 1:**
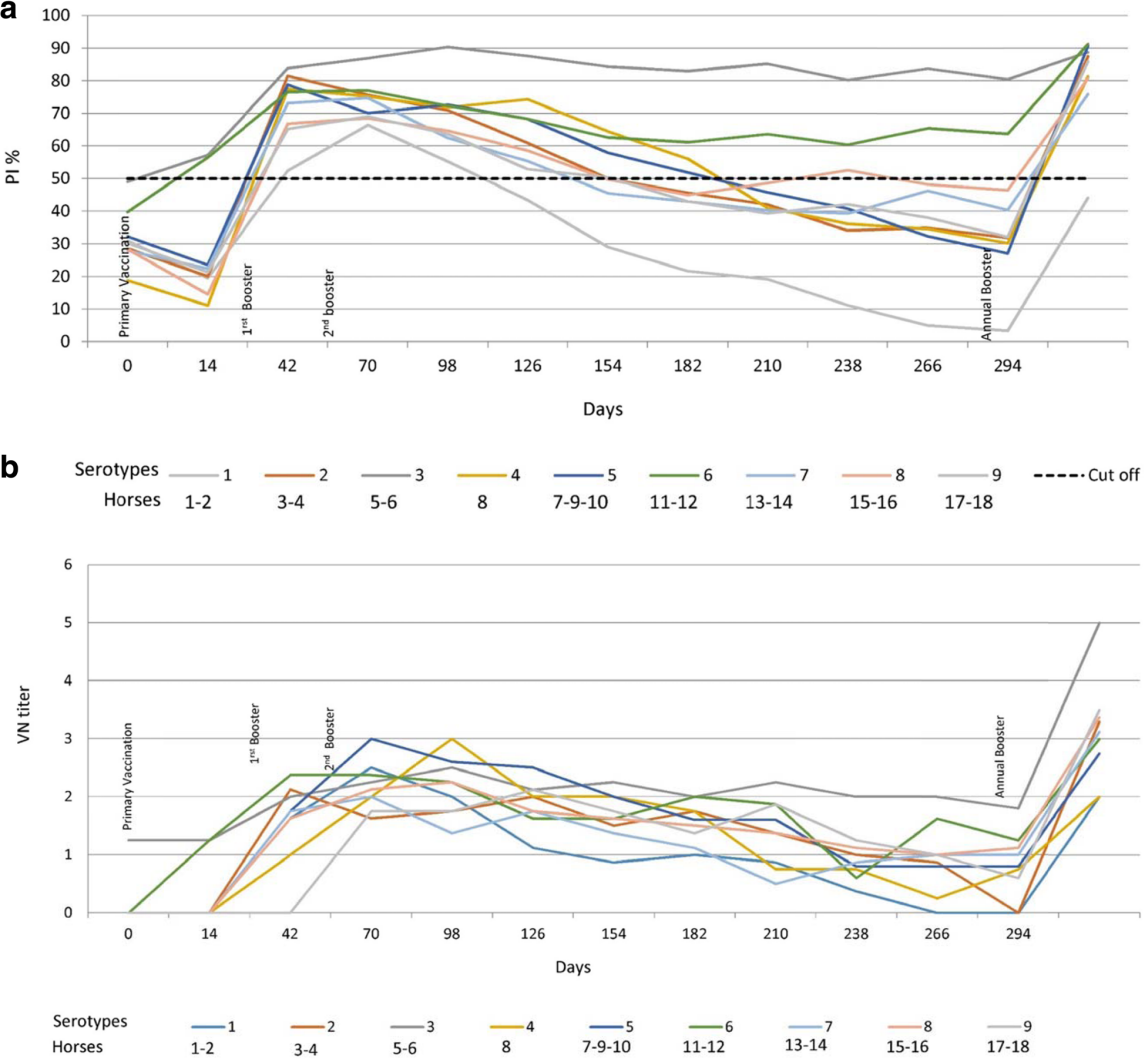
**a** Graphical representation of AHS ELISA antibody development in Group 1. Each serotype is the arithmetic mean of two horses. Horse 7 did not respond to serotype 4 and was revaccinated with serotype 5. Horse 1, died during the last trimester of the trial. **b** Graphical representation of AHS VN antibody development in Group 1. Each serotype is the arithmetic mean of two horses. Horse 7 did not respond to serotype 4 and was revaccinated with serotype 5. Horse 1, died during the last trimester of the trial

After primary vaccination, horses 5 and 11 demonstrated a rapid increase in antibody levels in both tests in comparison with the rest of the group. Two weeks after the first booster (day 42 pv /14 pb), 83% (15/18) of the horses seroconverted by cELISA and had a VN titre higher or equal to 1. After three vaccinations (day 98 pv/70 pb/42 2nd pb), 83% (15/18) of the horses remained positive by cELISA, whereas all horses (18/18) had a VN titre higher or equal to 1. Detailed results are shown in Table 1 for antibody development in the 18 horses against their assigned serotype. Figure 1(a, b) show the antibody development of serotypes 1 to 9 by cELISA and virus neutralisation test (VNT), respectively. Animal number 7 reacted neither to primary vaccination nor to the first or second booster with serotype 4. Therefore, this horse was revaccinated and boosted with serotype 5, and it became positive by both serological tests and was subsequently graded as a serotype 4 poor responder.

**Table 1:**
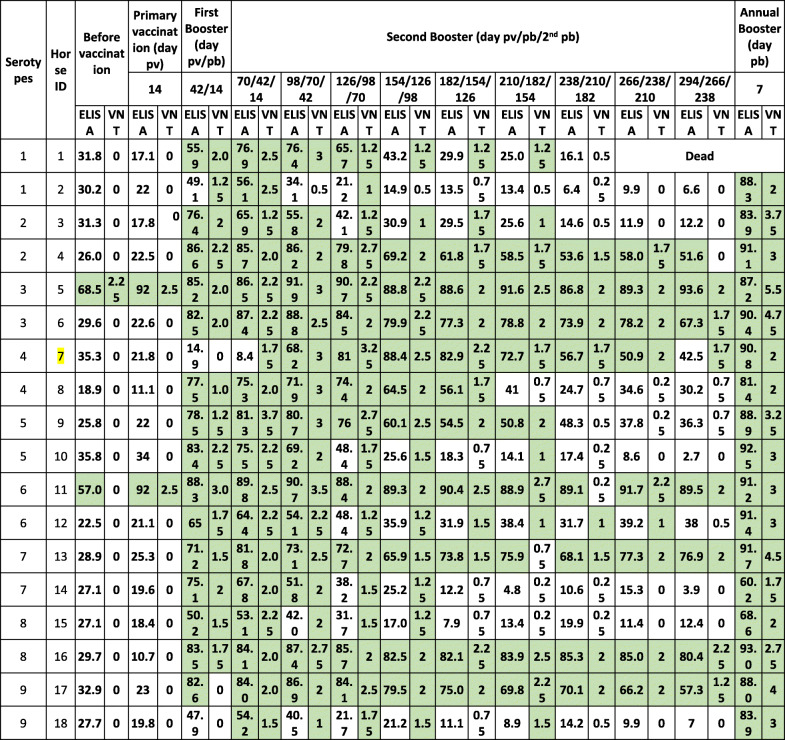
AHS ELISA* and VNT** antibody development in 18 horses after vaccination with single serotype vaccines (1–9) including 2 boosters

*pv* post vaccination, *pb* post booster, *2nd pb* second post booster

*ELISA is expressed as Percentage Inhibition (PI %) and Cut-off value for ELISA is ≥50% shown in green color

**VNT results are expressed as log 10 and titer ≥1 is shown in green color

Note: VNT was performed against respective serotype used in the vaccine

All horses remained serologically positive for 6 to 7 months, with the exception of horses 5 and 11, which remained antibody positive until the end of the experiment. The second booster did not significantly enhance antibody development; however, horses 17 and 18, which were vaccinated with serotype 9, developed neutralising antibodies only after the second booster. After 1 year, all 18 horses received their annual booster. Seven days after the annual booster, all horses seroconverted strongly. See Table 1 and Fig. 1(a and b).

### Group 2: antibody results in 11 horses vaccinated simultaneously with 9 serotypes

**Fig. 2:**
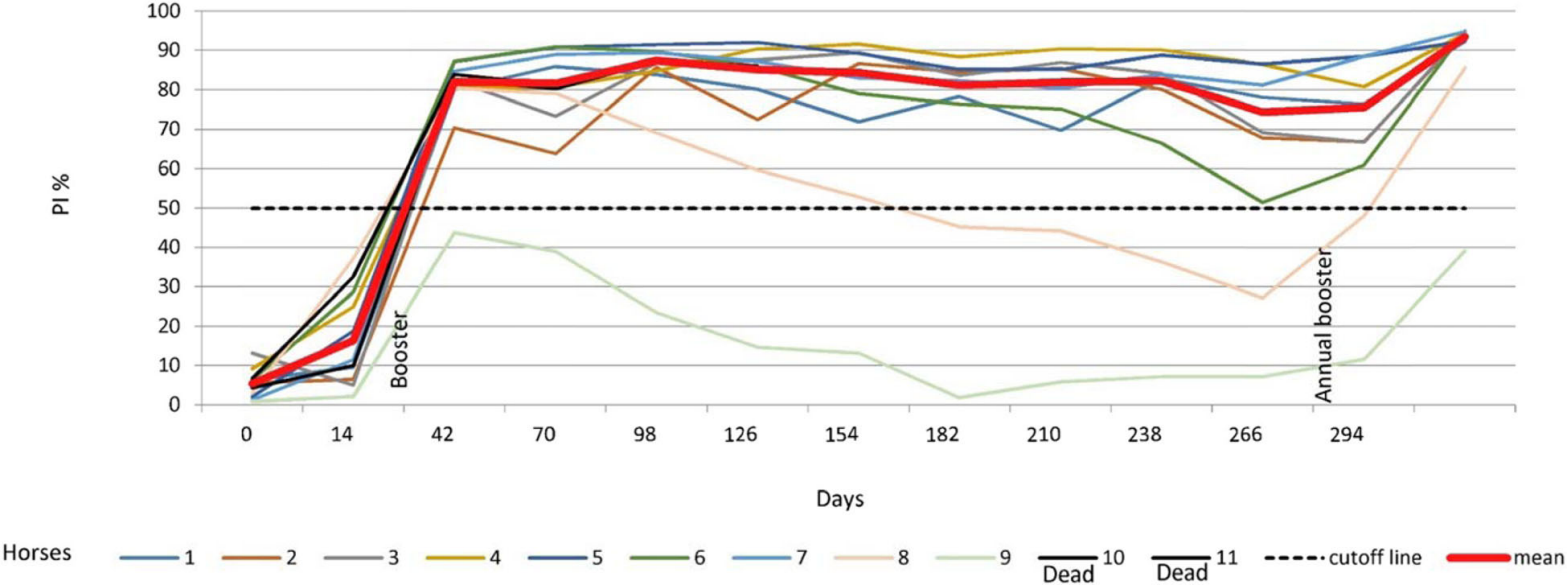
**a** Graphical representation of AHS ELISA antibody development in Group 2. Horses 10 and 11 died during the first trimester, due natural cause. 1. pv = post vaccination. 2. pb = post booster. 3. 2nd pb = second post booster. 4. ELISA is expressed as Percentage Inhibition (PI %) and Cut-off value for ELISA is ≥50% shown in green colour\. 5. VNT results are expressed as log 10 and titer ≥1 is shown in green colour

After primary vaccination, no antibody development was observed by cELISA, but antibodies above the cut-off level of 50% PI appeared between 5 and 14 days after the first booster (day 42 pv/14 pb) in 90% of the horses (10/11). Antibody levels remained stable until the end of the vaccination experiment, and 7 days after the annual booster, 8 out of the 9 horses seroconverted strongly. cELISA antibody development in each horse is presented in Fig. 2a.

The VN antibody results are presented in Tables 3, 4 and 5. VN antibodies, which started to increase in most horses before cELISA antibodies, which are not shown in Tables 3, 4 and 5, remained equal and/or above 1 until the end of the experiment for most of the serotypes. However, as shown in Tables 3, 4 and 5, all horses produced serotype-specific neutralising antibodies, but not always against all serotypes at the same time. Horse 9 was an cELISA poor responder, as the PI remained less than 50% throughout the trial but did produce VN antibodies, which were detectable until the end of the experiment (see Tables 2, 3, 4 and 5 and Fig. 2a).

**Table 2:**
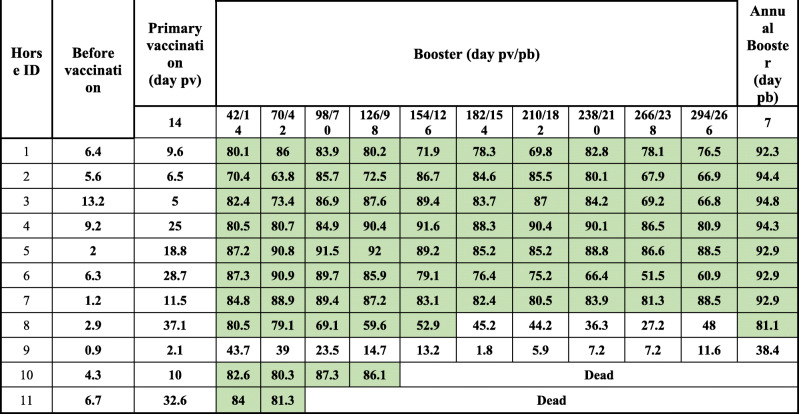
AHS ELISA* antibody development in 11 horses simultaneously vaccinated with all 9 serotypes in 2 shots including 1 booster

*pv* post vaccination, *pb* post booster

*ELISA is expressed as Percentage Inhibition (PI%) and Cut-off value for ELISA is ≥50% shown in green color

**Table 3:**
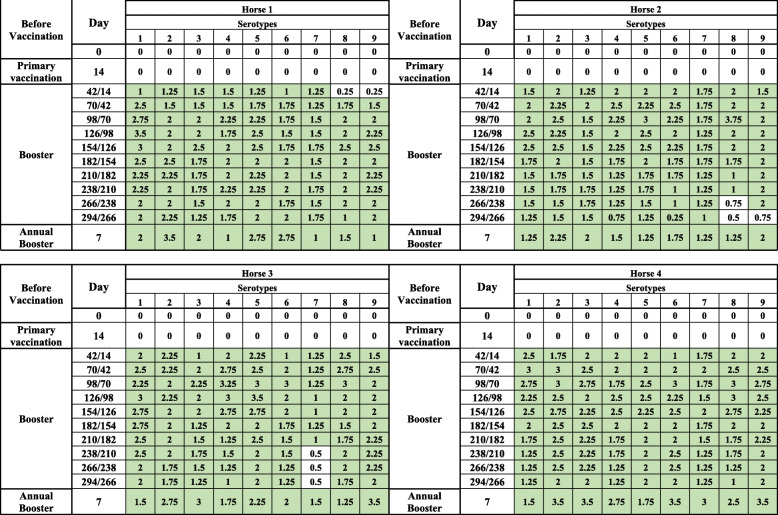
AHS VN antibody development in 11 horses simultaneously vaccinated with all 9 serotypes in 2 shots including 1 booster

VNT results are expressed as log 10 and titer is ≥1 shown in green colour

**Table 4:**
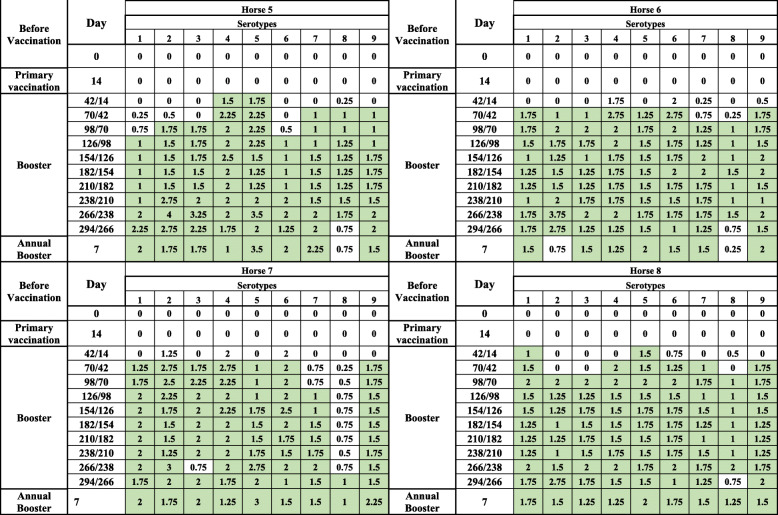
AHS VN antibody development in 11 horses after simultaneously vaccinated with all 9 serotypes in 2 shots including 1 booster

VNT results are expressed as log 10 and titer is ≥1 shown in green colour

**Table 5:**
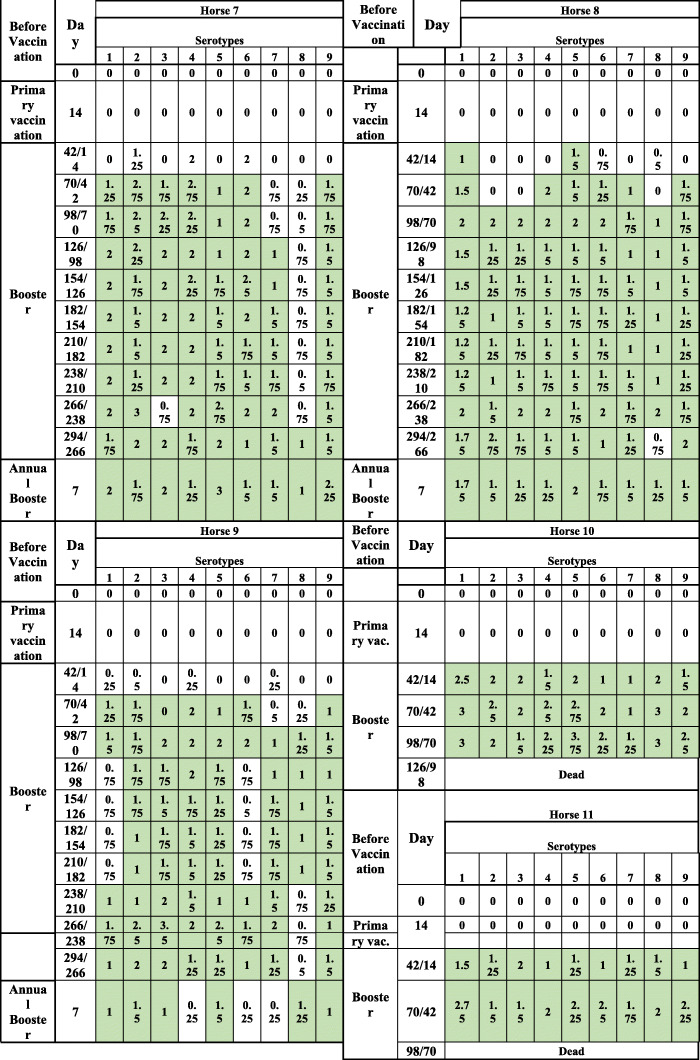
AHS VN antibody development in 11 horses simultaneously vaccinated with all 9 serotypes in 2 shots including 1 booster

VNT results are expressed as log 10 and titer is ≥1 shown in green colour

Observation of Group 1 (18 horses immunised with a single serotype 1 to 9) and Group 2 (11 horses simultaneously immunised with all 9 serotypes).

After each immunisation, some horses developed a minimal superficial lump at the injection site. Two horses developed warm swelling sized 10 to 11 cm. The swelling was treated twice a day with ice and receded after 4 days. Temperatures remained in the normal range for horses, between 37.2 °C to 38.3 °C. Horse 1 from Group 1 died during the last stage of the experiment, and horses 10 and 11 from Group 2 died 3 to 4 months after primary vaccination due to natural causes; therefore, the serological investigation could not be completed.

## Discussion

In total, 29 AHS horses over 20 years old, of different genders and kept in an isolated desert area in the Emirate of Dubai, United Arab Emirates (UAE), were immunised with inactivated AHS vaccines produced at the Central Veterinary Research Laboratory (CVRL), Dubai. Eighteen horses were immunised with individual AHS serotypes (two horses for each serotype), whereas 11 horses were simultaneously immunised with all 9 AHS serotypes in two formulations. All 9 serotypes were isolated from equine organs of horse fatalities in Kenya over a period of 17 years.

This vaccination experiment was performed because AHS has been found to occur in some of the vaccinated horses despite the use of attenuated vaccines [14, 17].

This study provides evidence that horses from Group 1, which were immunised with single serotypes (Table 1, Fig. 1a and b), were able to maintain cELISA and VN antibodies at high levels for only 5 to 7 months, which highly advises biannual vaccination. However, a second booster vaccination within a short period of time had no significant influence on antibody levels. It is worth mentioning that 2 horses vaccinated with serotype 9 developed cELISA and VN antibodies only after a second booster. Single AHS serotype vaccinations are necessary for controlling outbreaks where the specific serotype is known. AHSV RT-qPCR is proven to deliver accurate and fast serotype identification [8, 18] so that ring vaccination around the outbreak zone can start immediately or even on the same day when AHS vaccine banks, such as the one in Dubai, are available. Our results also showed that horses that had pre-vaccination cELISA titres caused by the OBP LAV reacted very fast to the inactivated CVRL vaccine, and their cELISA and VN antibodies remained high until the end of the experiment. Under current circumstances, it seems appropriate to use the OBP vaccine followed by an inactivated AHS vaccine [19] because we hypothesise that, in such instances, attenuated viruses or viral particles from the attenuated vaccine are neutralised by antibodies elicited by the killed vaccine, avoiding reassortment with a field virus. Horses immunised simultaneously with the 9 AHS serotypes in 2 vaccines seroconverted faster than horses in Group 1, and their cELISA and VN antibody titres remained detectable until the end of the trial in comparison to those in horses immunised with single serotypes. This indicates that immunisation with all 9 serotypes at the same time seems to have a synergistic effect on antibody production. Factors such as age and health of the animals or the nature of the vaccine itself could be the reasons for this synergistic effect. Simultaneous vaccination with inactivated polyvalent vaccines seems to enhance the immune response, which was not observed when attenuated polyvalent preparations were administered [20]. This is the first report demonstrating the immune response of horses to inactivated AHS vaccines containing all 9 serotypes. The European Medicines Agency [21] and several investigations [22, 23] have documented that inactivated Orbivirus vaccines are safe as the virus does not revert to virulence or cause viraemia in vaccinated animals or reassort with field Orbivirus strains.

There is no available treatment for AHS, and prevention can only be achieved by vector control and vaccination, which is a difficult approach, since all 9 serotypes can cause AHS. This is comparable to bluetongue virus (BTV), the prototype of the genus Orbivirus, which has morphology and characteristics identical to those of AHSV but with 28 serotypes.

In AHS-endemic countries, the temporal distribution of AHSV differs widely, and it is therefore unpredictable which serotypes circulate in a specific area [1, 24]. To protect horses against AHS in endemic countries, it is necessary to include all 9 serotypes in AHS vaccines, as there is generally no consistent cross protection between the serotypes. However, cross protection has been demonstrated between serotypes 5 and 8 and between serotypes 6 and 9 [4]. For example, Otieno and Amino [16], who investigated the distribution of AHS serotypes in Kenya, stated that in that country, horses should be vaccinated against all 9 serotypes, as all 9 serotypes have been isolated in Kenya.

Protection against AHS is serotype-specific, which means that horses must be immune to all 9 serotypes, and it is known that neutralising antibodies reflect immunity in horses [25]. However, to ensure polyvalent immunity against all 9 serotypes, horses need at least 3 to 4 annual vaccinations [11, 25].

The simultaneous administration of several attenuated AHS serotypes usually results in the production of antibodies against each serotype. However, the response of an individual horse to each serotype varies widely. The absence of detectable VN antibodies to one or more serotypes may not necessarily be suggestive of a lack of protection against AHS, as these animals might appear to be resistant to a challenge that also depends on cell-mediated immunity [26].

This situation is different when single serotypes emerge in non-endemic areas. In 1966, AHS serotype 9 entered Spain but was rapidly eliminated by vigorous single serotype vaccination and culling [27]. It is therefore essential for countries outside endemic AHS areas to establish AHS vaccine banks harbouring single inactivated vaccines to serotypes 1 to 9, which has been achieved by CVRL in the UAE for any emergency.

However, there are increasing concerns regarding the use of attenuated vaccines because of their potential reversion to virulence by re assortment of their gene segments with other vaccine and field serotypes, which was reported by Weyer and Weyer et al. [14, 17]. The authors performed disease surveillance using modern molecular techniques such as reverse transcription quantitative polymerase chain reaction (GSRT-qPCR) and genome comparisons confirmed that several AHS outbreaks in South Africa were either attributed to reversion to virulence of the attenuated vaccine strain serotype 1 or to a recombination of field and vaccine strains.

Similar drawbacks as those of attenuated Orbivirus vaccines have reported for attenuated BT vaccines, which may even cause abortion and congenital malformations when pregnant ewes are vaccinated. It has also been discussed that this disease may be caused in some sheep breeds by the vaccine virus itself with viremia in the vaccinated animals. This vaccine virus may then consequently be transmitted in the field by midges, thus coming in contact with field strains and undergoing reassortments to produce new virus strains. Consequently, the widespread use of such attenuated vaccines against BT was not recommended, and the recent BTV outbreak in Europe was controlled using inactivated vaccines [21, 22].

This led to the use of inactivated AHS vaccines [17] and the development of novel vaccines such as subunit vaccines and plant-based vaccines [28–30], thus avoiding these potential drawbacks. RNA fragments encoding the structural proteins VP2 and VP5 in the outer AHSV capsid, which are responsible for neutralising antibody production, can be inserted into different viruses, such as Baculovirus, vaccinia virus or capripox virus. During replication, these vectors express high quantities of proteins, which may then elicit protective immunity. However, establishing recombinant vaccines against all 9 AHS serotypes is time consuming and will require further investigations and financial support [29, 30]. Assuming that these new vaccines are 1 day commercially available, they may help not only reduce horse fatalities but also lift restrictions on the import and export of horses to and from endemic countries, as they may differentiate between vaccinated and naturally infected horses.

Due to the non-availability of a safe infection facility in the UAE, no challenge infection trial was performed after the 29 horses were immunised. However, 9 ponies that were vaccinated only once intramuscularly (im) with an inactivated AHS serotype 4 vaccine and then intravenously (iv) challenged with the same serotype all survived the challenge infection; only 3 of them had a brief period of fever (horse sickness fever), and only 1 of the 9 vaccinated ponies showed notable viraemia after challenge [23]. Similar cases of AHS fever were reported in Kenya, where more than 50 horses were recently simultaneously vaccinated with CVRL inactivated AHS vaccines containing all 9 serotypes in two injections. Several months later, six vaccinated horses showed mild clinical signs of AHS with swollen orbital, fever, increased heart rate and respiration. The horses had developed horse sickness fever, but they all survived, and the clinical signs receded within 72 h [31]. No live virus was isolated from their EDTA blood, but PCR was positive for serotypes 9 (4x), 4 (1x) and 1 (1x) when analysed at CVRL.

The regular use of inactivated AHS vaccines should protect against clinical signs and especially death. It is likely more difficult to prevent viremia in all vaccinated horses than to avoid infection of a vector. However, our investigations in Kenya showed that no live virus was isolated from vaccinated AHS cases with fever, but only AHSV RNA was detected, unlike cases reported by House et al. [23]. This situation must be more thoroughly investigated to further improve the inactivated vaccine. However, inactivated vaccines are optimal for immunising horse populations against AHS, as our experiment in Kenya showed, where in 2018/19, no AHS cases were reported (Spendrup, personal communication, 2019).

## Conclusion

CVRL AHS inactivated vaccines with 9 serotypes have been in production since 2014. These vaccines are available as individual serotype vaccines or vaccine combinations termed vaccine 1 with serotypes 1, 4, 7, 8, and 9 and vaccine 2 with 2, 3, 5, and 6. The serological results in 29 horses immunised with the CVRL inactivated vaccines show that horses immunised with individual serotypes need revaccination after 6 months and horses immunised simultaneously with all 9 serotypes after a year.

## Methods

### Cells

Baby hamster kidney 21 (BHK-21) from ATCC, Catalogue No. CCL-10™ passage number 53 were cultured in Minimum Essential Medium + Earle’s salts + L-Glutamine (MEM, Gibco, USA) supplemented with Fetal bovine serum (FBS, Gibco, Germany) while FBS was omitted for the cell virus replication. Cells were passaged twice per week in T75 flasks at a density of 4.5 × 10^5^ cells/ml and incubated in a humidified incubator at 37 °C with 5.0% CO_2_. BHK-21 cells were used to generate viral suspensions required to prepare the vaccines which was propagated in T300 flasks.

Vero cells from ATCC, Catalogue No. CCL-81™ passage number 120 were cultured with Minimum Essential Medium (MEM, Gibco, UK) supplemented with Fetal bovine serum (FBS, Gibco, Germany). Cells were passaged twice per week in T75 flasks at a density of 1.5 × 10^6^ cells/ml and incubated in a humidified incubator at 37 °C with 5.0% CO_2_.

Viral stocks were obtained by inoculating Vero cells in T75 tissue culture flasks. Also, infectious titer expressed in tissue culture infective dose (TCID_50_/ml) was determined with these cells, and virus neutralization tests were performed on Vero cells.

### Virus

The AHS viruses were isolated from unclotted whole blood, from lung lymph nodes as well as lung and spleen from dead animals originating from Kenya. The tissue samples were homogenised as a 10% (w/v) suspension in Minimum Essential Medium (MEM, Gibco, USA) containing 1% penicillin-streptomycin (Sigma Aldrich, Germany). The suspension was clarified by centrifugation at 2500 rpm for 5 min, and the supernatant was further diluted 1:10 in MEM. The diluted supernatant was sterile filtered with 0.45 μm filter (Sartorius, Germany) and inoculated into BHK 21 cells line grown in MEM. Three blind passages were performed for the presence of virus on BHK 21 cells and negative samples were further passaged 4 times on BHK 21 cells before considering as negative for the presence of the virus. Once the cytopathic effect (CPE) was observed, serotyping of the isolated AHS strains was carried out at the OIE AHS Reference Laboratories in Onderstepoort, South Africa; Madrid, Spain and at CVRL, Dubai, UAE. Each serotype was plaque purified on Vero cells by selection of large plaques (4–6 mm) at terminal dilutions. The plaque test was performed in Vero cells grown in 5 cm diameter petri dishes with an overlay of SeaPlaque Agarose 0.8% (Lonza, Rockland, ME, USA). The purification of AHS virus was carried out as described by Joklik [32] and Mirchamsy and Taslimi [33]. The final plaque material was passaged twice on Vero cells, and then tests for microbiological sterility including mycoplasma and extraneous viral agents of the stock virus were carried according to the guidelines of the OIE manual of diagnostic tests and vaccines for terrestrial animals [34]. Each serotype was freeze dried in 2 ml glass vials and frozen at − 80 °C. This master seed virus was resuspended in 2 ml sterile distilled water diluted with MEM and inoculated onto Vero cells to produce the working seed virus. From this, the viral suspension for the vaccine production was propagated in BHK 21 cells. The infectivity titers of each serotype were of each serotype were calculated before concentration and were found between 10^6.0^ and 10^7.5^ TCID50/ml.

Two to 3 days after infection, virus-containing cell culture supernatant was collected and concentrated 10 times by ultrafiltration using a Pelicon (R) 2 Mini Cassette (10KDa, Millipore, USA) filter.

The inactivation of the virus was performed as described by Ronchi et al. [35] apart from the addition of 37% formalin (Merck, Germany) to a final concentration of 1:8000 formaldehyde [36]. This was followed by the addition of 5 mM binary ethylenimine (BEI), the second inactivant, was prepared according to the method described by Bahnemann [37] by adding 1 N solution of 2-bromoethylamine hydrobromide (Sigma Aldrich B65705) to 0.175 N NaOH [37, 38]. Inactivation time varied from 25 h to 48 h for 300 ml of viral suspension based on the viral titres observed for each serotype. The inactivation process was stopped using 10% v/v 1 M sodium thiosulfate. All viral suspensions were stored at 2–8 °C. The inactivated virus solution was tested for residual activity by 2 different methods: the first method was passaging the inactivated viral solution 7 times through BHK 21 cells grown in T75 tissue culture flasks. The second method was passaging the virus 7 times into 9- to 11-day-old embryonated chicken eggs. The fluid from the final passages of both methods were tested by PCR for the detection of AHSV RNA.

### Real-Time PCR (RT-PCR)

This method followed the procedure laid down by Guthrie et al [18]., which is capable of detecting all 9 serotypes of AHS and is also prescribed in the OIE African horse sickness chapter [8]. RNA extraction was carried out from tissue culture supernatant, EDTA blood or tissue samples. Extraction was performed using the Magnapure automated extraction system and Magnapure total nucleic acid extraction kit (Roche, Switzerland). Extracted RNA was denatured at 95 °C for 5 min and frozen at − 20 °C for 5 min before use in RT-PCR. The total reaction volume was 25 μl, containing 5 μl of denatured RNA and 20 μl of TaqMan master mix with AHSV group-specific primer (concentration of 200 nM) and probe (concentration 120 nM), which was adapted from Guthrie et al. [17]. RT-PCR assays were performed on an ABI 7500 Dx RT-PCR instrument (Applied Biosystems, USA). The following thermal profile was carried out: 50 °C for 8 min, 95 °C for 2 min and 45 cycles of denaturation and annealing/extension at 95 °C for 15 s and 60 °C for 45 s, respectively.

Samples were considered positive if they showed an exponential amplification, a minimum fluorescence level of 0.1 and a cycle threshold of 36 or lower. Samples that amplified after this threshold were scored as weakly detected or negative based on repeated testing results.

### Serology

Two tests were used for the detection of AHSV antibodies, a cELISA that detects antibodies against VP7 and does not correlate with protection, and a VNT detecting antibodies against the surface antigens VP2 and VP5. The VNT is described in the OIE Manual of Diagnostic Tests and Vaccines for Terrestrial Animals [8] and approved by the European Commission [39].

The cELISA was performed according to Hamblin et al. [40] with in-house AHS antigen and anti-VP7 guinea pig sera.

The first step was the coating of all wells of the cELISA plate (Thermo Fisher, USA), with the in-house AHS antigen, which was diluted according to the optimal antigen strength in carbonate/bicarbonate buffer (Sigma Aldrich, Germany) at pH 9.6 and was incubated overnight at 4 °C. The following day, the plate was washed 3 times with phosphate-buffered saline (PBS) (Oxoid, UK) pH 7.6. The serum samples and negative control sera were diluted 1:5. The positive control sera which had a pre-determined titer was diluted across eight wells of the plate to give a final dilution of 1:640. All the samples and the control were in blocking buffer which contains, PBS, 0.05% Tween 20 (Sigma Aldrich, Germany), 5% skimmed milk powder (Sucofin, Germany) and 1% adult bovine serum (Gemini bioproducts, USA). Wells containing anti-VP7 guinea pig serum and blocking buffer were also included as a control for anti-guinea pig sera. The optimum dilution of each batch of anti-VP7 guinea pig antisera was pre -determined by checkboard titration. Diluted anti-VP7 guinea pig serum was added to all the wells and plate was incubated for 1 h and 15 min at 37 °C with shaking. After washing the plate 3 times with PBS, 1:1000 diluted conjugate (mouse anti-guinea pig horseradish peroxidase-labelled antibody (Dako, Denmark)) was added and the plate incubated for 1 h and 15 min at 37 °C with shaking. At the end of the incubation step, the plate was washed 3 times with PBS. Then, the substrate which was prepared by dissolving orthophenyldiamine tablet (4 mg, Sigma Aldrich, Germany) in 10 ml distilled water containing 0.005% of 30% hydrogen peroxide (Anala R, UK) was added and the plate was incubated for 10 min at room temperature in dark condition. The colour development was stopped by the addition of 1 M H_2_SO_4,_ (Ensure®, Germany) and the plate was read at 492 nm using an ELISA plate reader (Tecan Sunrise reader, USA) to obtain the optical density (OD). The interpretation of the results was based on percentage inhibition (PI), which was calculated as 100x (100-mean OD of sample/mean OD of anti-VP7 guinea pig control). Samples with PI values lower than 50% were considered negative, and samples with PI values greater than or equal to 50% were considered positive. The test was repeated for samples that were in the borderline range (PI = 49 to 50%).

Virus neutralisation: Serotype-specific antibodies against each serotype were tested using VNT according to Lelli et al. [41], Ronchi et al. [35] and OIE [8]. VNT was performed using isolated field strains, serotype-specific AHSV positive control antisera that were obtained from Pirbright Institute, UK. All test sera were inactivated at 56 °C for 30 min. In a 96-well flat-bottomed microtiter plate, 50 μl of 1:10 diluted sera in MEM were added. An equal amount of virus dilution of each serotype was added to 4 wells from 10^1^ to 10^7^. Positive and negative sera were included and the plates were incubated for 1 h at 37 °C with 5% CO_2_.

Vero cells at 5 × 10^5^ cells/ml were prepared in MEM + 10% FBS, and 100 μl were dispensed in each well. The test was read after 7 days of incubation. Virus titre was calculated using the Reed and Muench method. VN titres were derived by computing the differences between virus titres of each serotype in the presence of negative serum and the virus titres in the presence of the serum to be tested, which is expressed as log_10._

### Horses

Twenty-nine horses were included in the study with 25 gelding and 4 mares, aged between 20 and 30 years. Their history record was as follows: 13 were endurance horses, 8 thoroughbred and 8 sport horses. All horses were kept in a Desert Stud Stable. During the day, the horses were inside air-conditioned stables, and at night, they had access to open paddocks. Nutrition was provided twice daily in the form of GP mix, chaff, bran, hay, supplements (corn oil, electrodex, biotin, chevinal plus syrup, olive oil) and alfalfa. Unlimited access to water was also provided. The horses were divided into 2 groups. Group 1 comprised 18 horses that were subdivided into 9 subgroups of 2, and each pair was immunised with individual serotypes 1 to 9. In Group 2, 11 horses were simultaneously immunised with a combination of vaccine 1 and vaccine 2 (see below).

After the experiment ended, all the animals continued with their daily routine. No horse was euthanised.

### Vaccine/vaccination design/samples

#### Vaccine

The vaccine was formulated according to the manufacturer’s instructions with Imject Alum (Thermo Scientific, USA) as an adjuvant. The vaccines were presented in 2 forms, namely, single serotype vaccines and polyvalent vaccines administered in 2 formulations (vaccine 1 contained serotypes 1, 4, 7, 8, and 9, and vaccine 2 contained serotypes 2, 3, 5, and 6). In-house AHS antigen capture cELISA and PCR tests were employed to determine the concentration of each batch of the 9 monovalent vaccines. The antigen load was between 10^6.0^ and 10^7.5^ TCID50/ml. The virus concentration calculated for each serotype was the same for all three AHS vaccines, mono, quadrivalent and pentavalent.

All vaccines were manufactured and formulated prior to the start of the study. All vaccines were stored at 4–8 °C and were tested on horses for stability.

### Vaccination design

On Day 0, Group 1 and Group 2, horses were immunised as follows: Group 1: 2 ml of single serotype vaccines were sc administered into the left side of the neck. Group 2: 4 ml of vaccine 1 and vaccine 2 were sc administered into the left and right side of the neck, respectively.

On Day 28, Group 1 and Group 2 received a booster. On Day 56, Group 1 received a second booster. On Day 332, Group 1 and Group 2 received an annual booster.

### Samples

Blood samples were drawn from the jugular vein for cELISA and VNT every 2 weeks until the end of the trial, and blood was collected after each immunisation for 2 weeks (Days 0, 3, 7, 14) for PCR and virus isolation.

During the first 2 weeks after each immunisation, rectal temperatures were recorded twice a day, and the injection site was inspected.

## Data Availability

The datasets used and/or analysed during the current study are available from the corresponding author on reasonable request.

## Abbreviation

AHS: African horse sickness
AHSV: African horse sickness virus
BEI: Binary ethylenimine
BTV: Bluetongue virus
CPE: Cytopathic effect
CVRL: Central Veterinary Research Laboratory
cELISA: Competitive Enzyme Linked Immunosorbent Assay
Im: Intramuscular
Iv: Intravenous
MEM: Minumum Essential Medium
OBP LAV: Onderstepoort Biological Products Live attenuated vaccine Onderstepoort
OD: Optical density
PBS: Phosphate Buffered Saline
PI: Percentage of Inhibition
Pv: Post vaccination
Sc: Subcutaneous
VN: Virus neutralisation
VNT: Virus neutralisation test
UAE: United Arab Emirates
